# Assessing ResNeXt and RegNet Models for Diabetic Retinopathy Classification: A Comprehensive Comparative Study

**DOI:** 10.3390/diagnostics15151966

**Published:** 2025-08-05

**Authors:** Samara Acosta-Jiménez, Valeria Maeda-Gutiérrez, Carlos E. Galván-Tejada, Miguel M. Mendoza-Mendoza, Luis C. Reveles-Gómez, José M. Celaya-Padilla, Jorge I. Galván-Tejada, Antonio García-Domínguez

**Affiliations:** Unidad Académica de Ingeniería Eléctrica, Universidad Autónoma de Zacatecas, Jardín Juárez 147, Centro, Zacatecas 98000, Mexico; samaracosta@uaz.edu.mx (S.A.-J.); mauricio.mendoza@uaz.edu.mx (M.M.M.-M.); luiscarlosreveles@uaz.edu.mx (L.C.R.-G.); jose.celaya@uaz.edu.mx (J.M.C.-P.); gatejo@uaz.edu.mx (J.I.G.-T.); antonio.garcia@uaz.edu.mx (A.G.-D.)

**Keywords:** diabetic retinopathy, deep learning, convolutional neural network, ResNeXt, RegNet, SHAP

## Abstract

**Background/Objectives:** Diabetic retinopathy is a leading cause of vision impairment worldwide, and the development of reliable automated classification systems is crucial for early diagnosis and clinical decision-making. This study presents a comprehensive comparative evaluation of two state-of-the-art deep learning families for the task of classifying diabetic retinopathy using retinal fundus images. **Methods:** The models were trained and tested in both binary and multi-class settings. The experimental design involved partitioning the data into training (70%), validation (20%), and testing (10%) sets. Model performance was assessed using standard metrics, including precision, sensitivity, specificity, F1-score, and the area under the receiver operating characteristic curve. **Results:** In binary classification, the ResNeXt101-64x4d model and RegNetY32GT model demonstrated outstanding performance, each achieving high sensitivity and precision. For multi-class classification, ResNeXt101-32x8d exhibited strong performance in early stages, while RegNetY16GT showed better balance across all stages, particularly in advanced diabetic retinopathy cases. To enhance transparency, SHapley Additive exPlanations were employed to visualize the pixel-level contributions for each model’s predictions. **Conclusions:** The findings suggest that while ResNeXt models are effective in detecting early signs, RegNet models offer more consistent performance in distinguishing between multiple stages of diabetic retinopathy severity. This dual approach combining quantitative evaluation and model interpretability supports the development of more robust and clinically trustworthy decision support systems for diabetic retinopathy screening.

## 1. Introduction

Diabetic retinopathy (DR) is a serious ocular complication associated with diabetes mellitus (DM) characterized by damage to the retinal blood vessels. This disease can progress silently in its early stages, making early detection crucial to prevent irreversible vision loss. Worldwide, approximately 34.6% of diabetic patients are estimated to have some degree of DR, with 10.2% of these cases considered vision-threatening. In the United States, about 28.5% of adults with DM over the age of 40 are affected by DR [1,2]. The growing prevalence of DM worldwide underscores the urgency of addressing DR. According to the International Diabetes Federation (IDF), around 537 million adults were living with DM in 2021. Driven by socioeconomic, demographic, and lifestyle factors such as urbanization, an aging population, reduced physical activity, and increasing obesity rates, this figure is projected to rise to 783 million by 2045. Over 90% of these cases involve type 2 diabetes (T2D) [3]. This alarming trend highlights the importance of public health strategies aimed at mitigating the impact of diabetes and its complications, including DR. In addition to its ocular manifestations, the presence of DR is often an indicator of a heightened risk of complications in other organs, including the kidneys, heart, and brain [4]. For this reason, regular screening has been universally recognized as a critical element of comprehensive diabetes care. By identifying patients at risk of systemic complications, routine screening plays a pivotal role in improving overall health outcomes for individuals with diabetes. A fundus examination is the most reliable method for diagnosing DR. However, this process is labor-intensive and requires experts to manually identify specific retinal changes in each image, which can be both time-consuming and subject to variability among clinicians. This inconsistency can affect the accuracy of diagnoses, underscoring the need for more efficient and standardized approaches. An automated system for classifying DR severity could address these challenges by significantly improving the speed and uniformity of DR diagnoses and enhancing the management of vision problems associated with the disease [5]. The evaluation of DR through retinal imaging typically involves classifying the condition into five distinct grades: no diabetic retinopathy (NDR), mild non-proliferative DR (NPDR), moderate NPDR, severe NPDR, and proliferative DR (PDR) [6].

Artificial intelligence (AI) and deep learning (DL) algorithms are poised to play an increasing role in the coming decade, particularly in medical diagnosis, prognosis, and supporting management or treatment decisions [7,8,9]. Among the different DL techniques, convolutional neural networks (CNNs) have demonstrated exceptional performances in medical image analysis, particularly in tasks such as classification [10], segmentation [11], and anomaly detection [12]. CNNs are designed to automatically extract spatial hierarchies of features from input images, making them well suited for detecting intricate patterns associated with DR [13,14]. Their ability to learn from large-scale annotated datasets enables CNNs to achieve high accuracy in distinguishing between different severity levels, thereby reducing the reliance on manual feature engineering. Moreover, CNN-based models have been successfully integrated into automated DR screening systems, enabling rapid and standardized assessment of retinal images. Studies have shown that DL models can achieve diagnostic performance comparable to or even surpassing that of human experts in DR detection [15,16]. However, despite their high accuracy, one significant challenge associated with CNN-based DR classification is a lack of interpretability. The black-box nature of DL models raises concerns regarding their clinical adoption, as healthcare professionals require transparency in decision-making processes. To increase the transparency and interpretability of these models, explainable AI (XAI) techniques such as SHapley Additive exPlanations (SHAP) [17] have been implemented to elucidate the decision-making process of CNNs. SHAP enables the visualization of feature importance by identifying the specific retinal regions that influence a given classification. This enhances the interpretability of AI-based DR diagnosis, facilitating its integration into clinical practice by providing clinicians with transparent and justifiable model outputs.

The motivation for this research is to provide a comprehensive comparative analysis of ResNeXt [18] and RegNet [19] models for DR classification, evaluating their effectiveness in accurately detecting and grading DR severity. Given the increasing prevalence of DR and the limitations associated with manual grading, DL-based automated diagnostic systems have emerged as a promising solution to enhance efficiency, consistency, and accessibility in DR screening.

Moreover, this study utilizes SHAP to enhance the interpretability of the model’s decision-making process, allowing for the identification of key features. By increasing transparency, SHAP can facilitate the integration of AI-driven diagnostic systems into clinical practice.

The distinctive features and primary contributions of this study are outlined as follows:

- Comprehensive evaluation of ResNeXt and RegNet models: This study provides a detailed comparative analysis of the ResNeXt and RegNet architectures, focusing on their performance in DR classification.
- Integration of XAI with SHAP: To address the interpretability challenge in DL, SHAP is incorporated into the analysis. This approach enhances model transparency, providing clinicians with interpretable insights into the features that influence DR predictions.

### Related Work

Over the past several years, the scientific literature has extensively explored the application of DL techniques for the classification of DR. Numerous studies have evaluated a diverse array of CNN architectures, with a particular emphasis on enhancing diagnostic accuracy, computational efficiency, and model interpretability. This section provides an overview of recent advancements that address the classification of DR across both binary and multi-class tasks, incorporating various datasets, architectural modifications, and performance metrics.

The study of Mostafa et al. [20] investigated the application of DL for early prediction of DR, evaluating the performance of four CNN architectures: DenseNet201, ResNet50, VGG19, and MobileNetV2. Their objective was to determine the most efficient model for automated DR classification, striking a balance between accuracy and computational performance. The APTOS 2019 Blindness Detection dataset was used for training and evaluation. To assess model performance, the investigation employed validation accuracy, F1-score, precision, recall, and area under the curve (AUC). Among the standalone models, MobileNetV2 achieved the highest validation accuracy (78.22%), followed by DenseNet201 (76.98%), while ResNet50 and VGG19 underperformed, reaching 71.02% and 71.01%, respectively. The authors also explored an ensemble approach combining MobileNetV2 with Graph Convolutional Networks (GCN), which significantly improved performance. This hybrid model achieved 82.5% validation accuracy, an AUC of 0.88, an F1-score of 0.81, and a precision of 0.83, outperforming all individual CNN architectures.

Herrero et al. [21] evaluated the performance of multiple DL architectures for DR classification, including DenseNet, InceptionV3, MobileNetV3, ResNet50, VGG, and Xception. They also integrated SHAP to improve model interpretability. The ResNet50 model was modified through additional regularization techniques, fine-tuning, and TL to optimize its ability to distinguish DR severity levels and enhance generalizability across datasets. The models were trained and evaluated using five publicly available retinal image datasets: APTOS 2019 [22], EyePACS [23], DDR [24], IDRiD [25], and SUSTech-SYSU [26]. Model performance was assessed using accuracy, sensitivity, specificity, AUC, and Quadratic Weighted Kappa (QWK). Among the tested architectures, the modified ResNet50 demonstrated the highest overall performance, achieving 94.64% accuracy, an AUC of 0.98, and a QWK of 0.94 on the APTOS dataset.

The research of Kumar and Ravi [27] introduced a hybrid DL model for automated DR classification by combining a pretrained Inception-ResNet-v2 architecture with a custom CNN block. They evaluated their model on the APTOS 2019 and Messidor-1 [28] datasets, achieving accuracy of 82.18% and 72.33%, respectively. Their hybrid approach outperformed the baseline GoogleNet model, which reached only 66.03% accuracy on Messidor-1.

Ai et al. [29] constructed a holistic detection model called DR-IIXRN, consisting of a deep ensemble learning model integrating attention mechanisms for DR classification. This model combines five pretrained CNN architectures: InceptionV3, InceptionResNetV2, Xception, ResNeXt101, and NASNetLarge. The authors trained and evaluated the model using the Kaggle Diabetic Retinopathy dataset. Among the individual models, InceptionV3, InceptionResNetV2, and Xception all achieved accuracy of 0.77 or 0.78, with varying performance across different DR severity levels. NASNetLarge and ResNeXt101 exhibited slightly lower accuracy of 0.76, though both performed well in identifying severe DR cases. The DR-IIXRN ensemble model surpassed all individual models, particularly in no-DR classification (F1-score = 0.89) and proliferative DR (F1-score = 0.6).

Ramchandre et al. [30] explored a TL approach for DR classification utilizing pretrained DL models, namely, SEResNeXt32x4d and EfficientNetB3. The APTOS dataset was used for training and testing. Inputs were standardized in a preprocessing phase by resizing images, followed by data augmentation with AUGMIX to improve generalization. The EfficientNetB3 architecture outperformed SEResNeXt32x4d, achieving 91.44% accuracy compared to 85.16%, respectively.

Youldash et al. [31] explored the application of DL for DR, aligning with the Kingdom of Saudi Arabia’s Vision 2030 initiative, which promotes digital transformation in healthcare. Their research aimed to improve early detection and diagnosis of DR. The proposed approach utilized six pretrained CNNs: EfficientNetB3, EfficientNetV2B1, RegNetX008, RegNetX080, RegNetY006, and RegNetY008 to perform binary classification (DR vs no-DR) and multi-class classification (staging of DR severity). The study involved two experimental setups. The first experiment trained and evaluated the models using APTOS 2019, with the RegNetX080 model achieving the highest accuracy in binary classification (98.6%) and EfficientNetB3 obtaining the best performance in multi-class classification (85.1%). The second experiment trained the models on APTOS 2019 and evaluated them with fundus images from AI-Saif Medical Center in Saudi Arabia. In this setting, EfficientNetB3 achieved 98.2% accuracy in binary classification, while EfficientV2B1 reached 84.4% accuracy in multi-class classification.

With the rapid progress in DL and CNNs, automated DR screening has become a viable solution for large-scale clinical deployment. Table 1 provides a comprehensive and detailed comparison of state-of-the-art DL approaches used for DR classification, highlighting key aspects such as network architectures, datasets employed, top-performing models, and evaluation metrics.

## 2. Materials and Methods

**Figure 1:**
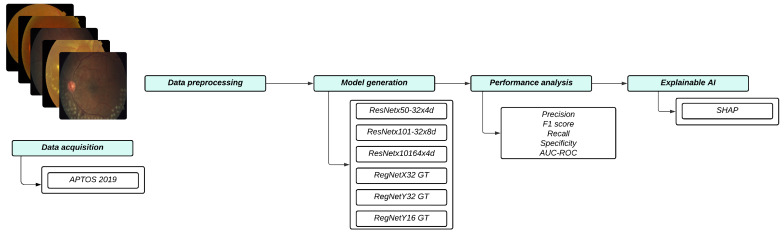
Workflow overview for the proposed DR methodology.

This study aims to compare DL architectures for classifying DR severity using the APTOS 2019—Blindness Detection Dataset. Figure 1 presents the workflow of the current study, outlining the steps from data acquisition to XAI.

### 2.1. Data Acquisition

Asia Pacific Tele-Ophthalmology Society (APTOS) 2019—Blindness Detection Dataset [22] contains fundus images categorized into five distinct classes: No DR (Class 0), Mild DR (Class 1), Moderate DR (Class 2), Severe DR (Class 3), and Proliferative DR (Class 4). The distribution of DR grades across the different data subsets (training, validation, and testing) is detailed in Table 2 for the multi-class classification task. This table shows the number of images in each class for the subsets. Additionally, Table 3 presents the data distribution for the binary classification task, where the goal is to distinguish between the presence and absence of DR. The original images provided by the APTOS 2019 challenge [22] have already been standardized to a resolution of 224x224 pixels, eliminating the need for additional resizing during preprocessing.

### 2.2. Data Preprocessing

Table 4 details the preprocessing steps implemented for the APTOS 2019 dataset. The Color Jitter transformation introduces random variations in brightness, contrast, saturation, and hue, enhancing the model’s robustness by simulating diverse lighting conditions. Random augmentations such as Random Horizontal Flip and Random Vertical Flip are employed to mitigate overfitting by artificially expanding the dataset. Additionally, Random Rotation rotates images within a 20-degree range, further diversifying the training samples. The Normalize transformation standardizes the pixel values based on predefined means and standard deviations for the RGB channels, promoting convergence during training. These preprocessing steps are applied to the training dataset, while only resizing and normalization are applied to the validation and test datasets. This distinction allows the evaluation metrics to accurately reflect the model’s performance on unaugmented real-world data.

### 2.3. Transfer Learning Models

This paper evaluates two state-of-the-art CNN architectures, RegNet [19] and ResNeXt [18], for transfer learning (TL) in DR classification. These architectures have been widely recognized for their high efficiency and accuracy in image classification tasks.

ResNeXt introduces a new dimension called cardinality, which enables improved feature extraction by increasing the number of parallel transformations; this architectural innovation significantly enhances the model’s representational capacity. Furthermore, the dual attention mechanism, which combines channel and spatial attention modules, allows this CNN to emphasize salient features while suppressing redundant information, thereby improving discrimination in complex visual scenes [32]. RegNet, on the other hand, is characterized by its design-automated architecture that harnesses a parameterized search space to generate efficient and scalable network topologies. It achieves high performance with fewer parameters, striking an optimal balance between computational efficiency and predictive accuracy [33,34]. These attributes make it particularly suitable for deployment in clinical scenarios where hardware limitations and inference speed are critical factors.

For both architectures, pretrained models trained on the ImageNet [35] dataset are employed to leverage their learned feature representations. The pretrained weights serve as an initialization point, allowing the models to capture low-level and high-level visual patterns relevant to retinal image analysis. The adaptation for DR classification involves fine-tuning the final layers, which optimizes the models for the specific task while preserving generalizable features derived from large-scale natural image datasets.

**Figure 2:**
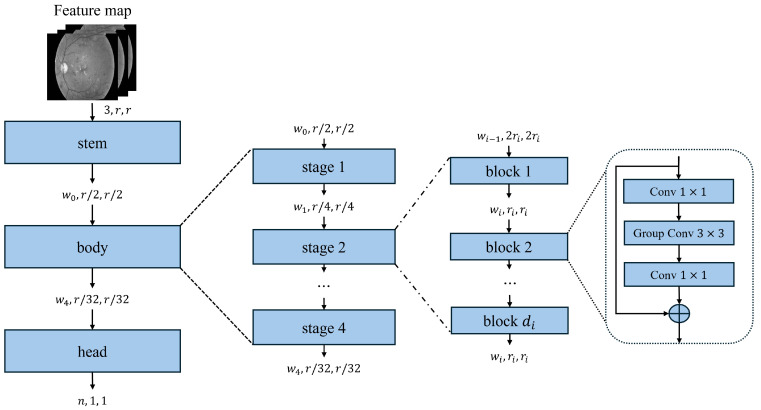
Block diagram of the RegNet architecture (adapted from [18]).

**Figure 3:**
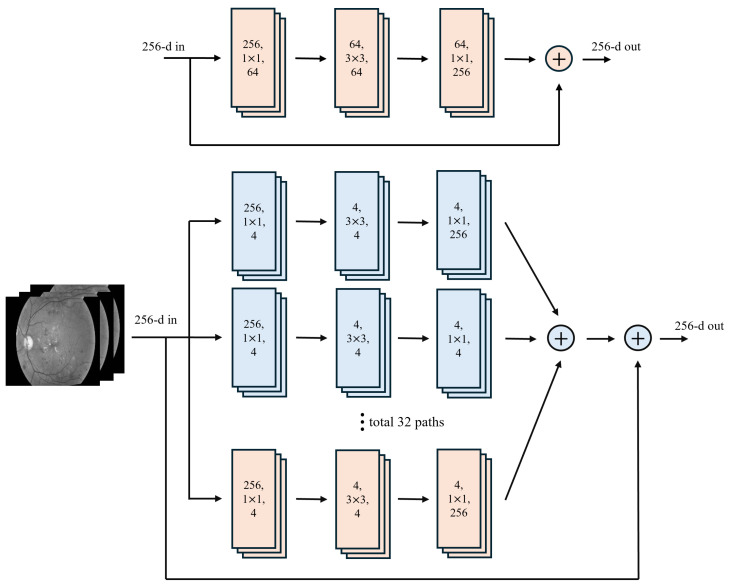
Block diagram of ResNeXt architecture (adapted from [36]).

- Regularized Network (RegNet) is a CNN model engineered to optimize architectural design through a parameterized framework, in contrast with traditional manually crafted architectures. Rather than relying on arbitrary design decisions, RegNet systematically determines the optimal depth, width, and group convolutions to maximize performance while maintaining computational efficiency (Figure 2).
  This network comprises two primary variants:
  – RegNetX: A less complex variant lacking additional transformations;
  – RegNetY: Integrates squeeze-and-excitation (SE) blocks while enhancing channel-wise feature recalibration, thereby improving feature representation and increasing accuracy.
  RegNet models follow a structured naming convention in which RegNetX/Y specifies the variant, while the (number) GT component represents the total number of floating-point operations (FLOPs) in giga-tiles (GT), indicating the computational complexity. The RegNet model family encompasses several notable variants. RegNetX-32GF is optimized for 32 giga-FLOPs, striking a balance between computational efficiency and task performance. RegNetY-16GF incorporates SE blocks, operating at 16 giga-FLOPs and enhancing feature recalibration. RegNetY-32GF is a more complex SE-augmented variant, utilizing 32 giga-FLOPs to refine feature extraction and classification capabilities further.

- ResNeXt: A deep CNN architecture that builds upon the ResNet framework, it introduces grouped convolutions and aggregated transformations. This network utilizes a cardinality parameter that denotes the number of parallel transformation pathways. This architectural design enhances representational capabilities while maintaining a manageable parameter count. By employing split–transform–merge operations, ResNeXt improves feature extraction without substantially increasing the parameter count (Figure 3).
  The ResNeXt models follow a structured naming convention in which
  (1) ResNeXt−[Depth]−[Cardinality]x[Width]
  specifies the architectural parameters; depth refers to the total number of layers in the network, cardinality represents the number of parallel transformation groups, and width defines the number of channels per group.
  – ResNeXt variants include ResNeXt-50-32x4d, a 50-layer model with 32 transformation groups, each with a width of 4, offering a well-balanced tradeoff between accuracy and computational efficiency. ResNeXt-101-32x8d is a deeper variant that retains 32 transformation groups but increases the width to 8, enhancing its ability to capture more complex feature representations. Meanwhile, ResNeXt-101-64x4d expands the number of transformation groups to 64, resulting in a higher-capacity model optimized for intricate classification tasks.

#### Computational Complexity

To complement the architectural description, Table 5 summarizes the computational complexity of the employed CNN architectures. These characteristics are particularly relevant for real-world deployment scenarios, where hardware limitations and latency requirements play a critical role. Of the selected CNNs, ResNeXt50-32x4d stands out for its relatively low complexity, with the fewest parameters, lowest GFLOPs, and fastest interference time, making it well suited for resource-constrained environments. In contrast, ResNeXt101-32x8d and ResNeXt101-64x4d offer deeper and wider configurations, resulting in higher parameter counts and computational demands; however, they are expected to deliver improved feature extraction due to their increased capacity. On the RegNet side, RegNetX32GF and RegNetY32GT exhibit the highest GFLOPs and memory footprints among the different architectures, reflecting their design for high-capacity feature representation. RegNetY16GF, which balances SE-block enhancements with moderate complexity, may serve as a middle-ground option between lightweight and high-capacity architectures. Notably, RegNetY16GT and RegNetY32GT require over 1.7 GB and 2.3 GB of GPU memory, respectively, which may limit their use in edge devices or low-resource clinical settings.

### 2.4. Model Generation

Establishing an effective training pipeline requires meticulous consideration of various parameters to ensure that the model converges to optimal performance. The training process begins with loading the dataset and executing preprocessing steps, which are crucial for enhancing the model’s ability to generalize across various image conditions as well as for mitigating potential biases.

The model training process is configured to ensure optimal performance (Table 6). The dataset is partitioned into three subsets: 70% for training, 20% for validation, and 10% for testing. The Adam optimizer [37] is employed with an initial learning rate of 0.001. This optimizer is selected due to its adaptive learning rate capabilities, which facilitate expedited convergence by adjusting the learning rate based on the gradients of the loss function. The cross-entropy loss function is employed for both multi-class and binary classification problems, offering a reliable metric to assess classification performance and ensure that the models effectively differentiate between various classes. A batch size of 32 images is used, striking a balance between memory usage and computational efficiency. Finally, each experiment runs for a total of 30 epochs, allowing ample time for the models to learn from the data and converge to an optimal solution.

### 2.5. Model Evaluation

The evaluation process assesses the model’s performance using a comprehensive set of metrics, including accuracy, precision, recall, F1-score, and AUC-ROC for both the multi-class and binary classification tasks. These metrics offer a holistic view of the model’s effectiveness across various dimensions of classification.

(2) $$Accuracy=\frac{TP+TN}{TP+TN+FT+FN}$$

(3) $$Precision=\frac{TP}{TP+FP}$$

(4) $$Recall=\frac{TP}{TP+FN}$$

(5) $$Specificity=\frac{TN}{TN+FP}$$

(6) $$F1-score=2\cdot \frac{Precision\cdot Recall}{Precision+Recall}$$

Accuracy provides a general measure of overall correctness by quantifying the proportion of the true results among the total number of evaluated cases. Precision and recall provide insights into the performance concerning positive predictions, while specificity evaluates the model’s ability to correctly identify negative cases. The F1-score is the harmonic mean of the precision and recall, offering a balance between these two metrics, which is especially useful in scenarios with class imbalance. Finally, AUC-ROC further illustrates the tradeoffs between true positive rates and false negative positive rates, contributing to an understanding of the model’s classification capabilities [38].

### 2.6. Model Interpretability

To improve the transparency and interpretability of DL models, SHAP is integrated as an explainability technique. SHAP provides a quantitative assessment of each input feature’s contribution to a model’s prediction, making it a fundamental tool for interpreting complex machine learning (ML) models. Rooted in cooperative game theory, SHAP calculates the marginal contribution of each feature by systematically considering all possible combinations. It allows for a more comprehensive and theoretically grounded attribution of feature importance [39]. Each SHAP value can be positive or negative, with positive values indicating an increase in the likelihood of a particular classification and negative values indicating a decrease. In the context of DR image analysis, individual pixels are treated as features, and SHAP generates visual explanations in heat maps. These heat maps highlight the regions that most influence the model’s decision, with red areas indicating high-contribution regions towards a specific classification and blue areas denoting features that do not significantly impact the decision-making process.

## 3. Results

### 3.1. Binary Classification Performance

Table 7 summarizes the binary classification performance of the ResNeXt and RegNet models. Overall, all architectures demonstrated high discriminatory capability in distinguishing DR (Class 1) from non-DR cases (Class 0), as evidenced by elevated AUC, precision, sensitivity, specificity, and F1-score values across both classes.

Among the ResNeXt variants, the ResNeXt101-64x4d model yielded the strongest overall results, achieving an AUC of 99.71% for both classes, precision of 96% and 99%, sensitivity of 99% and 96%, and F1-score of 98% for both classes. This performance reflects the model’s capacity to effectively minimize both false positives and false negatives.

Within the RegNet family, the RegNetY32GT architecture yielded the most consistent and well-balanced classification results, with an AUC of 99.31% for both classes, a precision and sensitivity of 97%, and a specificity value of 97.25% and 96.76% for Class 0 and Class 1, respectively. These results position RegNetY32GT as the top-performing model in its group, combining precision with robustness.

**Figure 4:**
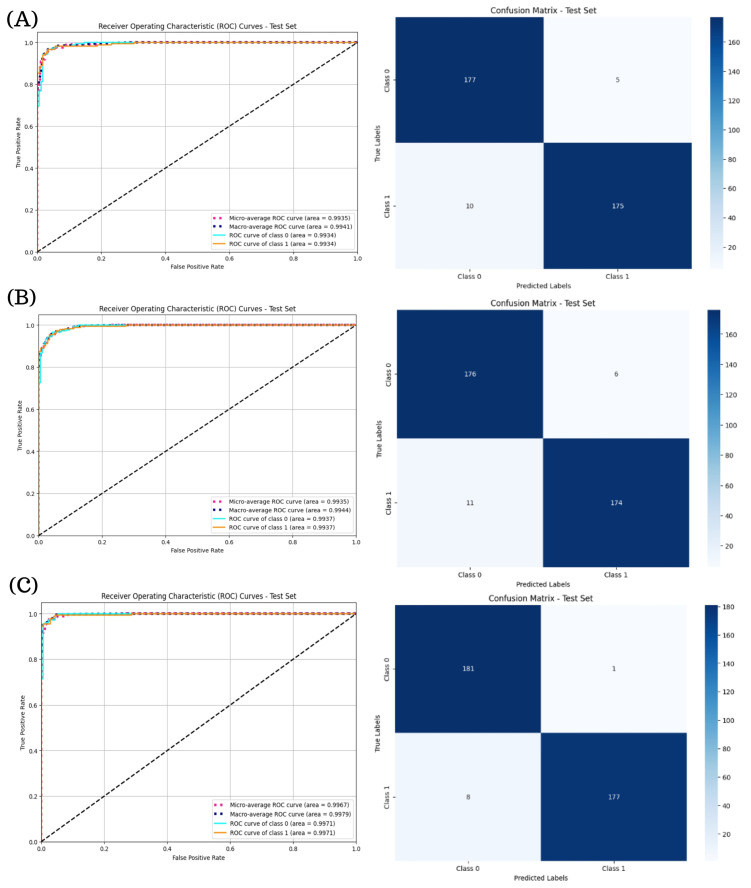
ROC curves and confusion matrices for ResNeXt models in binary DR classification. Subfigure (**A**) corresponds to the ROC curve and confusion matrix for ResNeXt50-32x4d, subfigure (**B**) presents the ROC curve and confusion matrix for ResNeXt101-32x8d, and subfigure (**C**) shows the results for ResNeXt-101-64x4d.

Figure 4 illustrates the ROC curves and confusion matrices for the ResNeXt models. In particular, ResNeXt101-64x4d (Figure 4C) achieved near-perfect performance, with micro- and macro-average AUCs of 0.9967 and 0.9976, respectively, and only nine misclassifications on the test set. The shallower ResNeXt-50-32x4d and intermediate ResNeXt101-32x8d (Figure 4A,B) also performed reliably, but with lower AUCs (∼ 0.9934) and greater number of errors (15 and 17, respectively).

**Figure 5:**
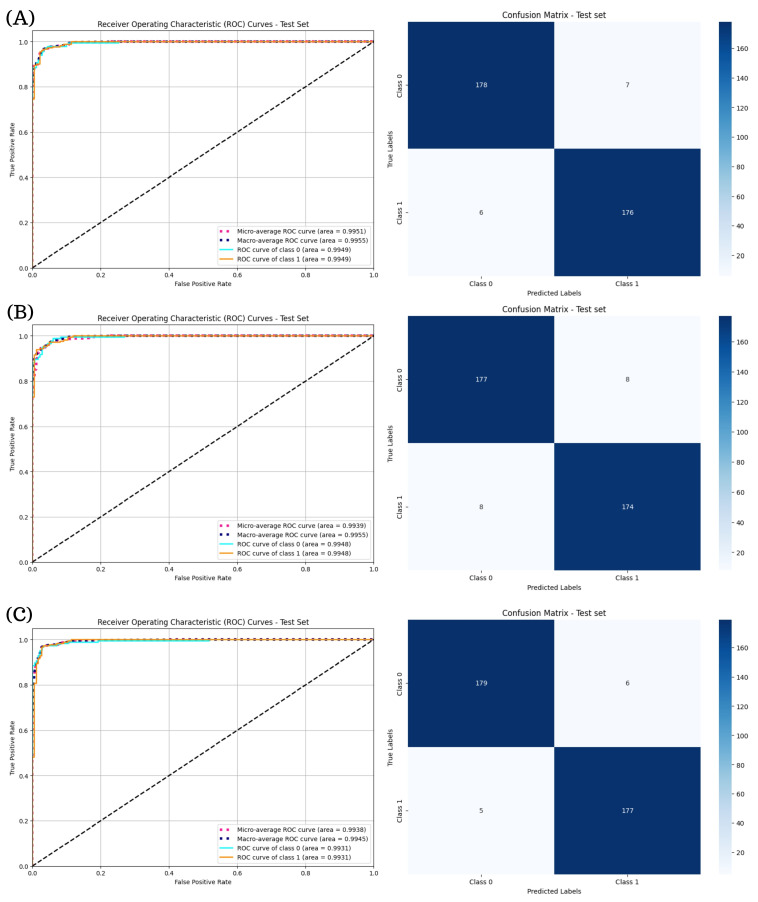
ROC curves and confusion matrices for RegNet models in binary classification. Subfigure (**A**) depicts the ROC curve and confusion matrix for the RegNetX32GT model, subfigure (**B**) shows the ROC curve and confusion matrix for the RegNetY16GT model, and subfigure (**C**) shows the results for the RegNetY32GT model.

Correspondingly, Figure 5 presents the ROC curves and confusion matrices for the RegNet models.

The RegNetY32GT model (Figure 5C) achieved the most favorable results, with micro- and macro-average AUCs of 0.9938 and 0.9945, respectively, and the lowest total number of misclassifications. Both RegNetX32GT and RegNetY16GT (Figure 5A,B) also maintained high performance, with macro-average AUCs above 0.994 and minimal false predictions, particularly in Class 1.

While binary classification shows excellent performance, it represents a simpler scenario where the task is limited to detecting the presence or absence of DR. Conversely, the multi-class setting poses more substantial clinical and computational challenges, as well as the propensity of models to misclassify moderate and severe stages. The subsequent section examines how these same CNN architectures perform when tasked with grading DR severity across five distinct classes.

### 3.2. Multi-Class Classification Performance

Table 8 summarizes the multi-class classification results obtained by the ResNeXt and RegNet architectures. Across all models, Class 0 consistently achieved the highest performance, characterized by superior AUC, precision, sensitivity, and F1-score values. In contrast, performance declined notably in the moderate to severe DR stages (Classes 1–4), reflecting the greater difficulty in distinguishing between similar pathological features in advanced cases.

Regarding the ResNeXt family, the ResNeXt101-32x8d model proved to be the most consistent and well-balanced across all classes. In Class 0, it achieved an AUC of 99.84%, precision of 96%, sensitivity of 98%, and F1-score of 97%, demonstrating excellent discrimination of healthy cases. For Class 2, it maintained strong performance (AUC = 92.56%, F1-score = 78%), outperforming other ResNeXt variants in this category. Although performance dropped in Classes 3 and 4, it still yielded F1-scores of 42% and 48%, respectively, which are higher than those of the shallower (50-32x4d) and broader (101-64x4d) configurations. This indicates that the deeper32x8d) architecture allows for better representation while maintaining efficiency.

**Figure 6:**
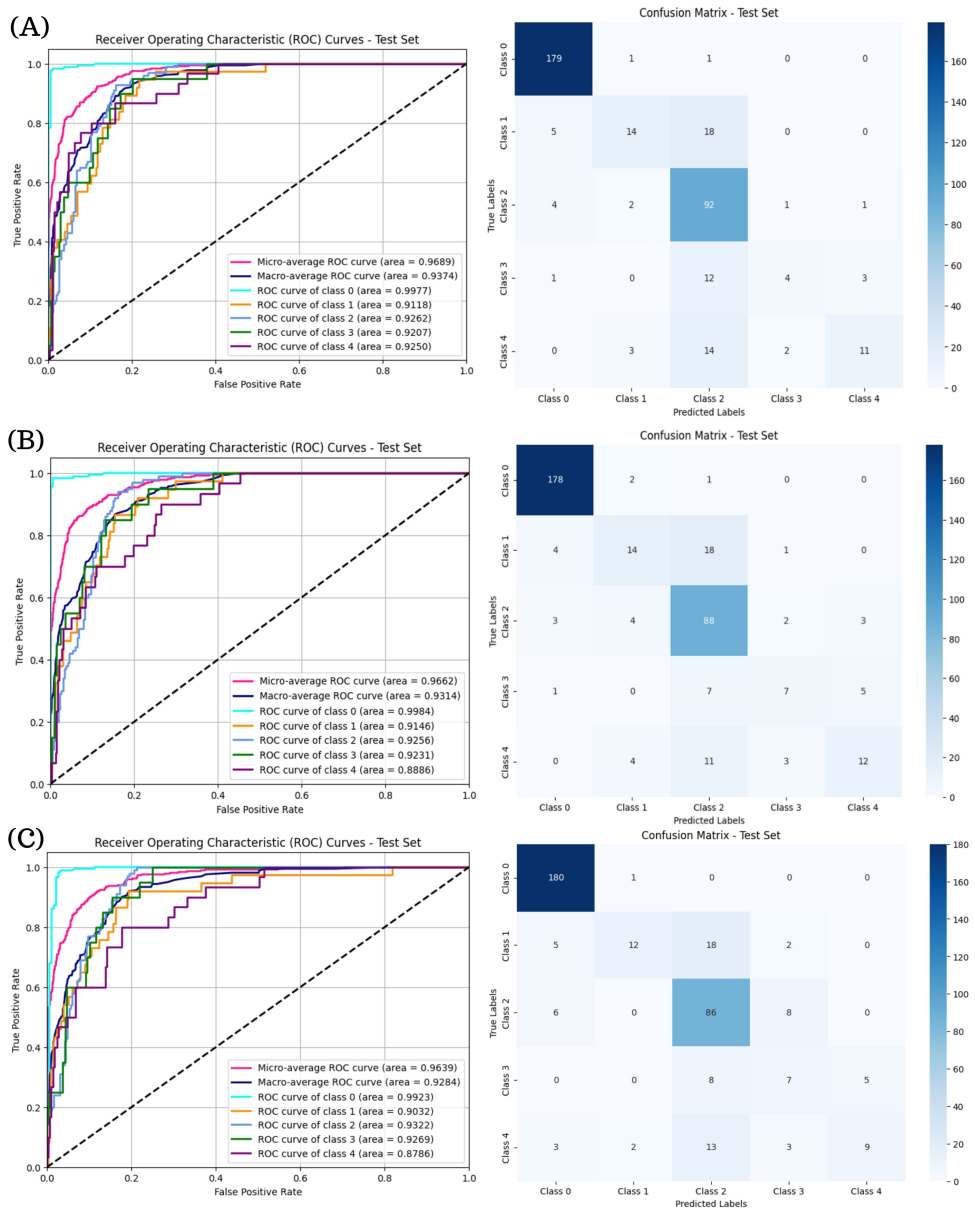
ROC curves and confusion matrices for ResNeXt models in multi-class DR classification. Subfigure (**A**) corresponds to the ROC curve and confusion matrix for ResNeXt50-32x4d, subfigure (**B**) presents the ROC curve and confusion matrix for ResNeXt101-32x8d, and subfigures (**C**) shows the results for ResNeXt101-64x4d.

Figure 6 illustrates the ROC curves and confusion matrices for the ResNeXt models in multi-class DR classification. The ResNeXt101-32x8d model (Figure 6B) provided the most favorable balance across DR stages, attaining a micro-average AUC of 0.9662 and macro-average AUC of 0.9314 as well as the most consistent class-wise ROC curves. While ResNeXt50-32x4d and ResNeXt101-64x4d (Figure 6A,C) also achieved high AUC values, their confusion matrices reveal greater misclassification rates in the moderate and severe DR categories.

**Figure 7:**
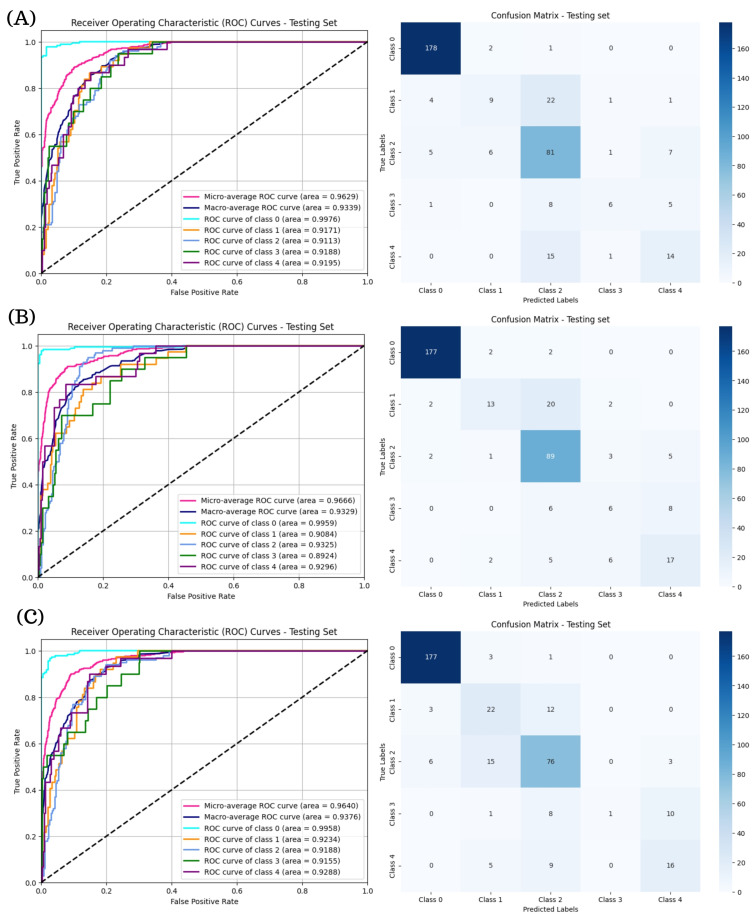
ROC curves and confusion matrices for RegNet Models in multi-class DR classification. Subfigure (**A**) presents the ROC curve and confusion matrix for the RegNetX32GT model, subfigure (**B**) illustrates the ROC curve and confusion matrix for the RegNetY16GT model, and subfigure (**C**) displays the results for RegNeY32GT model.

The ROC curves and confusion matrices presented in Figure 7 reveal that RegNetY16GT demonstrates the strongest overall performance among the RegNet models evaluated for multi-class DR classification, achieving a macro-average AUC of 0.9376 and exhibiting consistent class-wise separability, particularly in Classes 0 and 2. Although RegNetX32GT and RegNetY32GT also attained high AUC values, their confusion matrices indicate increased misclassification rates in Classes 3 and 4. In contrast, RegNetY16GT showed more balanced predictions across all severity levels, suggesting a better capacity to generalize across varying DR stages.

Turning to the RegNet family, RegNetY16GT showed the most balanced performance across all severity levels. It achieved an F1-score of 98% in Class 0 and attained the highest F1-score in Class 2 (80%), with a sensitivity of 89%, highlighting its reliability in detecting moderate DR. For Class 4, it achieved an F1-score of 57%, surpassing RegNetY32GT, which scored 54%. Although performance in Class 3 remained limited, RegNetY16GT still outperformed its RegNetX32GT counterpart, indicating superior generalization under challenging categories.

To synthesize the overall performance trends observed in the models, Table 9 outlines the top-performing models from the ResNeXt and RegNet families. In the binary DR classification setting, the ResXt104-64x4d architecture consistently surpassed its counterparts, achieving perfect or near-perfect metrics across all evaluated categories. Its F1-score of 98% and AUC of 99.71% indicate outstanding predictive stability. In the case of the RegNet group, the RegNetY32GT variant demonstrated the most reliable behavior, with uniformly high values in precision, sensitivity, and specificity confirming its robustness in distinguishing DR from non-DR cases.

Performance variability was more apparent in the multi-class scenario. ResNeXt101-32x8d achieved a leading AUC of 99.84% and exhibited strength in accurately identifying Class 0 and Class 2 instances. Conversely, RegNetY16GT showed enhanced resilience across a broader range of DR stages, particularly Classes 2 and 4, where it produced the highest F1-scores among all evaluated models. Its ability to maintain consistent sensitivity across multiple severity levels underscores its potential for more granular DR staging. These findings emphasize not only the importance of model selection based on task complexity but also the architectural influence on inter-class discrimination.

### 3.3. Shapley Additive Explanations

To improve the interpretability of the classification results, the top-performing models from the ResNeXt and RegNet families were selected based on their outstanding performance in both binary and multi-class DR tasks, then further evaluated using SHAP. This selection was informed by the quantitative results summarized in Table 9, ensuring that the subsequent explainability analysis focused on the architectures with the highest predictive consistency and robustness. By applying SHAP, the analysis aimed to uncover the most impactful retinal areas influencing the model’s predictions, thereby fostering a more transparent and clinically meaningful decision-making process.

### 3.4. SHAP-Based Interpretability for Binary DR Classification

**Figure 8:**
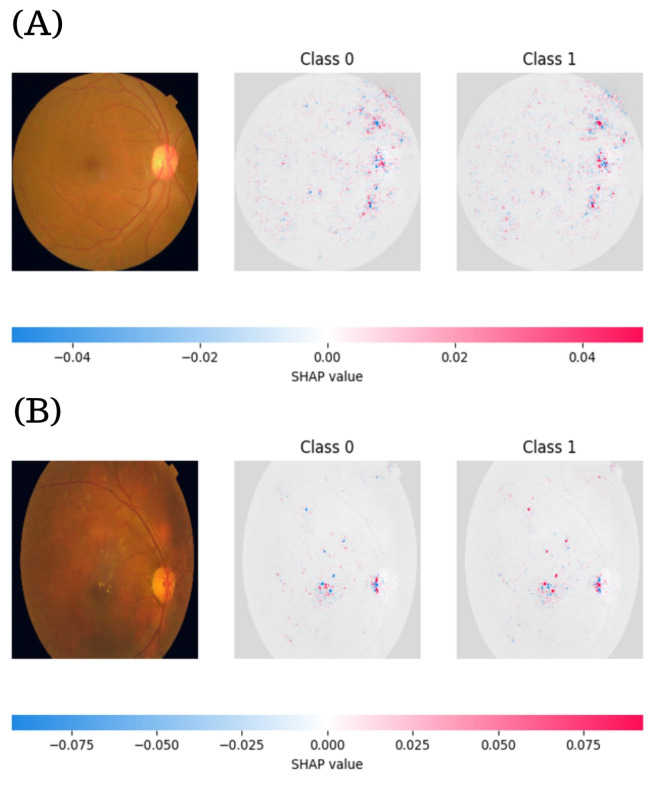
SHAP visual explanations for binary DR classification using the ResNeXt101-64x4d model. (**A**) Healthy fundus image with corresponding SHAP maps for Class 0 and Class 1. Red regions indicate features that support the prediction, while blue regions represent opposing contributions. In the healthy case, the model’s attention is concentrated around the optic disc and major vessels, with minimal activation in the Class 1 map. (**B**) Fundus image showing DR; the SHAP maps highlight pathological features such as microaneurysms, hard exudates, and hemorrhages, particularly near the macula and along vascular structures.

Figure 8 presents the SHAP visualizations for ResNeXt101-64x4d, which was the top-performing model on the binary classification task. The color maps illustrate the pixel-wise contribution values for Classes 0 and 1, providing insight into the model’s decision-making process. The examples include a healthy fundus image (Figure 8A) and an image exhibiting signs of DR (Figure 8B). Each subfigure comprises the original fundus image followed by SHAP heatmaps corresponding to Class 0 and Class 1 predictions.

In the healthy case (A), the SHAP values range from −0.04 to 0.04. The model’s attention is predominantly localized around the optic disc and vascular structures. The red regions in the Class 0 map highlight features that positively contributed to a “no DR” classification, whereas blue regions indicate features that slightly contradicted this prediction. Conversely, the Class 1 map displays a reversed color gradient, with red and blue tones suggesting limited activation and supporting the model’s confidence in ruling out DR. Importantly, the peripheral areas of the retina remain largely inactive in both maps, suggesting that the model concentrates its attention on the central fundus, avoiding noisy or non-discriminative regions.

In the DR case (B), the SHAP values extend to a broader range (−0.075 to 0.075), denoting more pronounced contributions from pathological features. In the Class 0 map, strong blue activations indicate areas that counter a healthy classification. In contrast, the Class 1 map shows intense red zones around the macula and along large vessels, regions that are typically associated with DR-related lesions such as microaneurysms, hemorrhages, and hard exudates. A notable observation is that the Class 0 and Class 1 maps are not simple inverses of each other; instead, they emphasize distinct and complementary features, indicating that the model forms independent reasoning pathways for each class rather than relying on binary negation.

**Figure 9:**
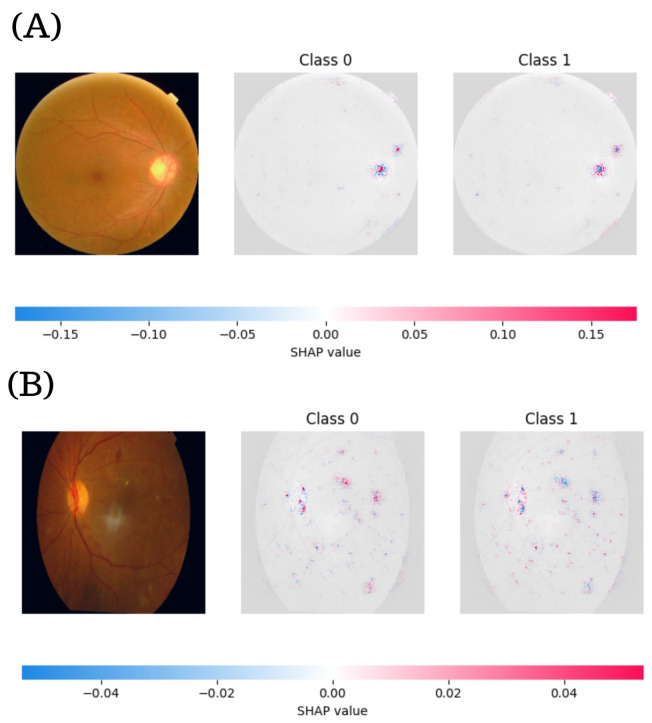
SHAP visualizations using the RegNetY32GT model. (**A**) For the healthy fundus image, the SHAP maps reveal focused activations around the optic disc and main vessels, with symmetrical class-wise patterns indicating stable interpretation of normal anatomical features. (**B**) In the DR case, the SHAP responses shift toward pathological regions, with intense red zones highlighting lesion-specific features.

Figure 9 displays the SHAP visualizations for the RegNetY32GT model, which was selected as one of the most robust performers in binary DR classification. In the healthy case (A), the original fundus image reveals normal retinal anatomy, characterized by a visible optic disc and regular vascular branching. The SHAP value scale extends from −0.15 to 0.15, reflecting substantial model activation even in the absence of disease. In the Class 0 map, positive contributions (red areas) are concentrated in the optic disc, denoting that this region plays a key role in the model’s validation of a non-DR classification. Conversely, blue zones indicate minimal contradiction to that outcome. Class 1 shows a mirrored distribution, with blue tones emphasizing non-pathological characteristics. The symmetric spatial pattern across both classes, particularly around the optic disc and large vessels, highlights the model’s stability and consistency in processing healthy anatomical features.

In contrast, the DR-positive case (B) exposes pathological signs such as scattered hard exudates and vascular abnormalities. Here, the SHAP values range from −0.04 to 0.04, indicating a more localized and nuanced 1interpretive scope. In the Class 0 map, negative contributions are more diffusely distributed, signaling that various retinal regions challenge the classification as healthy. Class 1 shows concentrated red zones, particularly near the macula and along primary vascular paths. These areas correspond to lesions typical of DR. Notably, the Class 1 SHAP map reveals more focal and lesion-specific activation compared to Class 0, reinforcing the model’s ability to isolate clinically meaningful features while discounting noise.

### 3.5. SHAP-Based Interpretability for Multiclass DR Classification

**Figure 10:**
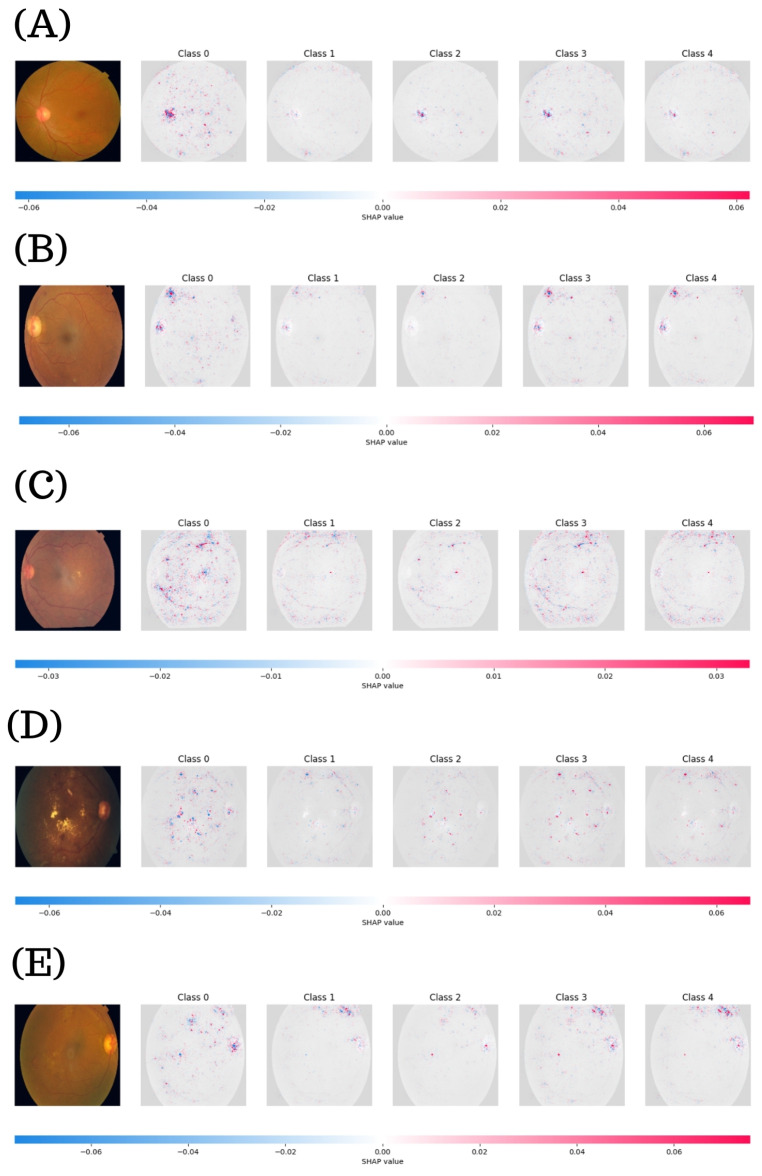
SHAP visualization explanations for multi-class classification using ResNeXt101-32x8d. Each row (**A**–**E**) corresponds to a test image representing a specific DR severity level (Classes 0 to 4). Columns display the original fundus image alongside SHAP maps for each class output.

Figure 10 displays the SHAP maps generated by the ResNeXt101-32x8d model for five representative test cases (A–E), each associated with one of the five DR severity levels. Each subfigure includes the original fundus image and the corresponding SHAP maps for Classes 0 through 4. The color map represents feature contributions to each class-specific prediction, where blue tones indicate inhibitory effects and red tones denote supportive contributions. In case A (Class 0), the fundus image presents no apparent retinal damage (consistent with the predicted label). SHAP values range between -0.06 and 0.06. The map highlights localized red activations, primarily around the optic disc and macular region while the other class maps remain nearly neutral, reinforcing the model’s confidence in excluding early or advance DR.

Case B (Class 1) shows a subtle deviation from normality in the original image. The SHAP maps reveal faint activations across all classes, with slightly stronger responses in Class 4. This suggests that although the ground truth is mild DR, the model identifies structural cues that partially align with more severe cases. Notably, the Class 1 map shows minimal activation, which may reflect the model’s limited sensitivity to early-stage DR changes or its tendency to conflate subtle features with those of higher grades.

In case C (Class 2), the SHAP values span a narrower interval (−0.03 to 0.03) and activations appear more uniformly distributed across Classes 1 to 3. This dispersion suggests that the model identifies overlapping characteristics between moderate and adjacent severity levels, which aligns with the clinical challenge of differentiating between borderline DR stages. The spatial distribution of red and blue areas suggests that the model assigns greater weight to mid-retinal structures than to peripheral cues for Class 2 predictions.

For case D (Class 3), the image shows well-defined exudates and vascular irregularities. The SHAP maps confirm these pathological markers, with intense activations in Class 3 and Class 4. These lesions are consistently highlighted in red across both classes, supporting the model’s attribution of these features to advanced DR. The pronounced contrast between Classes 0–2 and Classes 3–4 reflects a more binary interpretation of DR progression at this stage. Finally, case E (Class 4) details widespread retinal damage; SHAP maps exhibit dense regions in Class 3 and Class 4, particularly near hemorrhagic sites and neovascular formations. This distribution confirms that the model focuses on clinically significant abnormalities to justify its severe classification. Additionally, the lack of red activations in Classes 0-2 reinforces the model’s capacity to discount irrelevant features in advanced cases.

**Figure 11:**
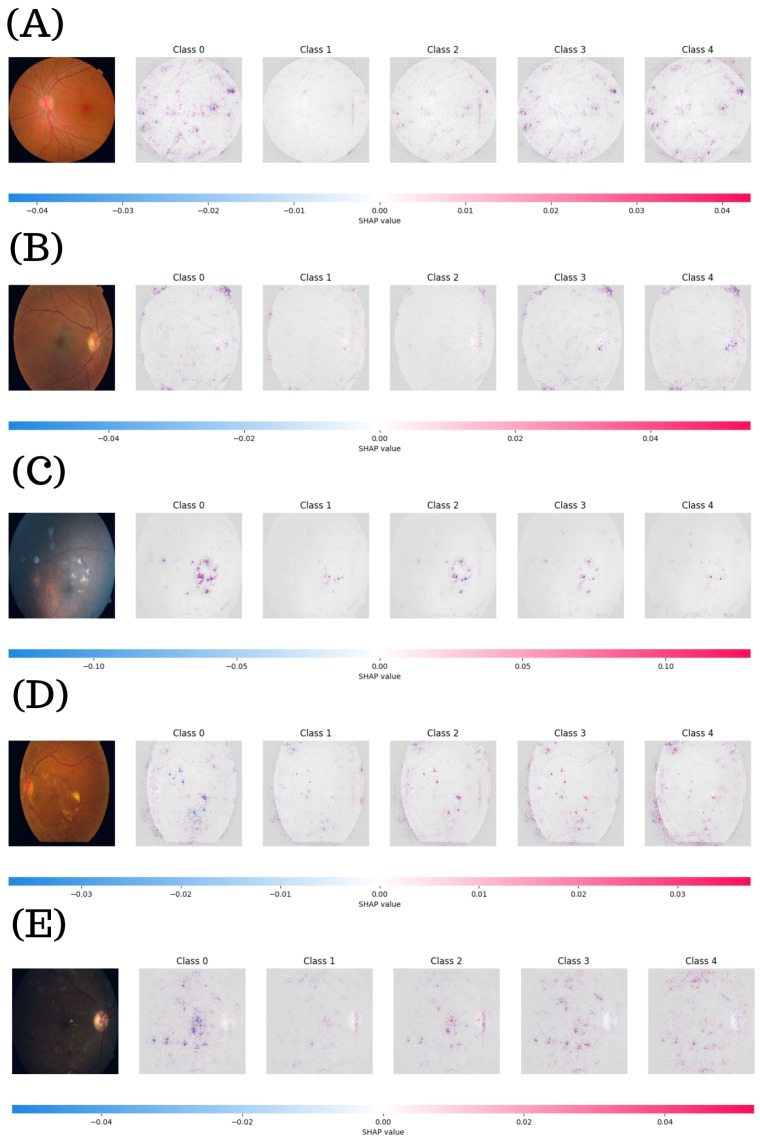
SHAP visualization explanations for multi-class classification using RegNetY16GT. Each case (**A**–**E**) corresponds to a different DR severity level, ranging from Class 0 to Class 4.

Figure 11 shows the SHAP visualizations generated by the RegNetY16GT model. In case A, the fundus image appears anatomically normal, with a SHAP range from −0.04 to 0.04. The model focuses on physiological landmarks such as the optic disc and major vessels as key contributors to the non-DR classification. Interestingly, adjacent class maps (e.g., Class 1 and 2) exhibit weak but structurally similar activations, suggesting that the model retains a baseline understanding of anatomical continuity across stages.

Case B (Class 1) presents mild retinal alterations. The red regions are concentrated around early lesion indicators, and the interclass SHAP maps reveal more precise boundaries between diagnostic stages, indicating enhanced class separation at early pathological thresholds. In case C (Class 2), the SHAP values expand to a range of −0.10 to 0.10, reflecting a more decisive model response. The red activations in Class 2 are pronounced and spatially focused, particularly around the macula. The presence of activation contrast between Class 2 and Classes 1 and 3 suggests that moderate DR exhibits improved discriminative sensitivity, possibly due to the more apparent manifestation of lesions. Case D (Class 3) exhibits moderate DR features, including exudates. The SHAP range is narrower (−0.03 to 0.03) with red and blue activations diffusely scattered across multiple class maps. The overlap in SHAP intensities among Classes 2 to 4 suggests classification ambiguity. Lastly, case E (Class 4) reports severe pathology, with SHAP values ranging from −0.04 to 0.04. Class 4 displays dense and well-defined red regions corresponding to key pathological areas. The symmetry of SHAP responses across Classes 3 and 4 reflects the model’s ability to contextualize and escalate its classification with disease severity.

## 4. Discussion

Grading the degree of DR is of great significance in treating patients, since timely and accurate classification enables early intervention and prevents vision loss. Given the increasing prevalence of DR worldwide, the development of reliable automated classification systems has become a pressing need in ophthalmic screening programs. In this work, a comprehensive evaluation was conducted comparing two advanced DL architectures, ResNeXt and RegNet, in terms of their capacity to classify fundus images in both binary (DR vs non-DR) and multi-class (five-stage severity) settings. The experimental design involved standardized training procedures, performance assessment through multiple metrics, and model interpretability analysis using SHAP. This dual approach not only ensured robust quantitative evaluation but also provided insight into the decision-making process of each model. The study aimed to assess the discriminative power, generalization behavior, and clinical transparency of these models across various DR stages.

We ensured consistency across evaluations by training all models under identical experimental settings, partitioning the dataset into 70% for training, 20% for validation, and 10% for testing. In the binary task, the objective was to distinguish between healthy retinas and those exhibiting any signs of DR, regardless of severity. Table 7 summarizes the performance of the ResNeXt and RegNet models based exclusively on predictions over the independent test subset. When comparing the performance of both families, notable differences emerged in terms of metric balance, model depth, and architectural efficiency.

The ResNeXt101-64x4d configuration yielded the most favorable results overall, achieving an AUC of 99.71% for both classes, precision and sensitivity of 99%, and an F1-score of 98%, which outperformed all other models in the ResNeXt and RegNet groups. This superior outcome can be attributed to its deeper and wider architecture, which enhances the model’s capacity to capture subtle retinal features while maintaining stability during training. The cardinality aspect of ResNeXt (grouped convolutions) allows for a richer representation of complex patterns, which is especially beneficial when differentiating fine-grained characteristics between healthy and diseased retinas. On the other hand, the RegNetY32GT model showed remarkable consistency, achieving identical AUC values (99.31%) for both classes and uniform scores (97%) across precision, sensitivity, specificity, and F1-score. This homogeneity suggests robust generalization and fewer outlier-driven predictions. The strength of the RegNet family lies in its highly regularized design and block-wise optimization, which promotes architectural simplicity without sacrificing performance. Unlike deeper ResNeXt configurations that rely on large parameter counts, RegNet models achieve competitive accuracy through parameter efficiency and well-calibrated depth–width tradeoffs. From a practical standpoint, while ResNeXt101-64x4d demonstrated marginally higher scores in most metrics, RegNetY32GT offers a more lightweight and computationally efficient alternative, making it attractive for deployment in resource-constrained clinical settings or real-time screening systems. Additionally, complementing our quantitative evaluation, SHAP visualizations for the ResNeXt101-64x4d model revealed strong and focused activations around clinically relevant anatomical structures.

In healthy cases, the models concentrated their attributions on retinal vessels and the optic disc nerve head, exhibiting narrow ranges of values. Conversely, DR-positive predictions emphasized lesion-related regions such as microaneurysms and exudates, displaying broader and more intense activations. Furthermore, the models demonstrated clear class-specific attribution patterns; ResNeXt101-64x4d produced distinct non-overlapping regions between Class 0 and Class 1. At the same time, RegNetY32GT consistently highlighted early DR features such as localized hemorrhages, despite operating within a narrower SHAP value range. This behavior supports the interpretability and diagnostic alignment of both models.

Building upon the binary classification analysis, the multi-class configuration posed a more complex challenge, requiring the models to differentiate among the five clinical stages of DR progression. it is worth mentioning that both families of models demonstrated remarkable multi-class classification capabilities. However, the evaluation of class discrimination revealed some disparities in performance (Table 8).

The ResNeXt101-32x8d architecture achieved the highest AUC for Class 0 (99.84%) and demonstrated robust performance in the intermediate DR stages, notably in Class 2, with an 88% sensitivity and F1-score of 78%. This outcome reflects the model’s architectural depth and grouped convolutions [40], which facilitate the extraction of discriminative features in well-represented classes. However, its performance declined for advanced DR stages, particularly Class 4, where sensitivity dropped to 40%, likely due to data imbalance and increased intra-class variability. In addressing class imbalance, it is important to note that the present study did not apply specific mitigation strategies such as oversampling, class weighting, or focal loss during training. The models were intentionally evaluated under the natural distribution of the APTOS 2019 dataset in order to reflect real-world clinical screening scenarios. Even so, the performance drop in advanced DR stages, particularly in Classes 3 and 4, highlights the limitations imposed by underrepresented categories. This imbalance reduces the ability of models to learn discriminative features for rare but clinically critical cases. In contrast, RegNetY16GT exhibited more balanced and stable outcomes across all stages, with AUCs exceeding 89% and a notably higher sensitivity in Class 4 (57%), accompanied by consistent F1-scores. These results can be attributed to RegNet’s network regularization strategy and optimized connectivity patterns [41], which enhance generalization in cases of complex retinal pathology. Thus, while ResNeXt excels in early-stage DR discrimination, RegNetY16GT appears better suited for recognizing severe cases, offering improved reliability in real-world screening settings where detecting high-risk patients is essential.

To further examine the interpretability of the top multi-class models, SHAP maps from ResNeXt101-32x8d and RegNetY16GT were utilized to reveal distinct attribution patterns that aligned with DR severity. Specifically, ResNeXt101-32x8d showed a clear progression in SHAP intensity, transitioning from focused activations on anatomical landmarks in early stages to widespread highlights over pathological regions in advanced DR. In contrast, RegNetY16GT displayed more evenly distributed contributions across severity levels, with class-specific heatmaps and reduced overlap, suggesting finer internal differentiation. Notably, in severe cases it was able to consistently identify multiple lesions, including macular damage and vessel abnormalities, showing enhanced interpretative transparency. These visualization outcomes complement the quantitative metrics, suggesting that the models may capture patterns aligned with clinical reasoning. However, further validation by ophthalmologists is needed to confirm the reliability of these interpretability maps.

To contextualize the observed results, it is essential to compare the performance of the proposed architectures with that of state-of-the-art approaches previously applied to DR classification. While numerous studies have reported high overall performance using various convolutional models and ensemble strategies, these are often presented as global metrics without detailed class-wise evaluation. This limits their clinical interpretability, particularly in settings where distinguishing between different stages of DR is crucial for effective treatment planning. A comparative analysis with the recent literature not only highlights the methodological advantages of this study, including per-class reporting, dual binary and multi-class evaluation, and explainability via SHAP, but also underscores its clinical relevance in the context of real-world screening applications.

In particular, Ayala et al. [42] employed a DL architecture with TL on the APTOS dataset, achieving notable performance in early-stage DR, with an F1-score of 0.59 for Class 1 and 0.77 for Class 2. However, their model exhibited a marked decline in later stages, with F1-scores of 0.31 and 0.40 for Classes 3 and 4, respectively. In contrast, the ResNeXt101-32x8d model tested in our study achieved more balanced and robust results, particularly in advanced stages, reaching F1-scores of 0.42 and 0.48 for the same grades. Additionally, it demonstrated higher recall and precision in Classes 2 to 4, which is critical for minimizing missed diagnoses in high-risk patients (Table 10).

Another study by Mohanty et al. [43] evaluated two models on the APTOS dataset. The first was a hybrid model, which achieved an accuracy of 79.50%, while the second was a DenseNet121-based model, which achieved a higher accuracy of 97.30%. Although heir DenseNet121 configuration attained strong global performance, no per-class breakdown or clinical interpretability was provided. In contrast, our RegNetY16GT model yielded an accuracy of 82%, surpassing their hybrid model and offering a more interpretable and balanced performance across DR grades. This distinction is critical, as stage-specific insights and consistent classification across DR severity levels are essential for clinical screening applications that require patient risk stratification.

R. Yasashvini et al. [44] contributed to APTOS-based research by implementing a conventional CNN architecture consisting of sequential Conv2D and MaxPooling layers followed by a flattening operation and dense layers. They trained the model over ten epochs with a small batch size, and evaluated its performance using the confusion matrix. Despite this methodological clarity, their CNN achieved an overall accuracy of only 75.61%, which falls short compared to the models evaluated in the present study (82% for RegNetY16GT and 81% for ResNeXt101-32x8d).

Lin et al. [41] implemented a revised ResNet-50 model for DR classification, reporting a test accuracy of 74.32%, which slightly outperformed the baseline ResNet-50 (75%) as well as other CNNs such as AlexNet (73.04%), VGGNet-s (73.66%), and VGGNet-16 (48.13%). Although the primary aim of their investigation was to evaluate a standard operation procedure (SOP) and visualize model behavior rather than to maximize classification performance, the reported results still indicate modest effectiveness. In comparison, the models in our study achieve accuracy levels of 82% and 81%.

Regarding ensemble-based approaches, Ai et al. [29] proposed DR-IIXRN. They reported an overall accuracy of 79% for their model, with class-wise F1-scores of 0.89 for non-DR and 0.60 for PDR. In contrast, the present study offers a more comprehensive evaluation using RegNetY16GT and ResNeXt101-32x8d architectures on the APTOS dataset, yielding not only higher overall accuracy (82% and 81%, respectively) but also consistently high AUC values (above 92% in most classes).

Finally, while this study has provided a comprehensive evaluation of ResNeXt and RegNet architectures, two limitations must be acknowledged. First, despite the robust performance we observed in both binary and multi-class settings, notable variability in sensitivity and F1-scores was revealed, most specifically in advanced DR stages. This discrepancy can be attributed to class imbalance within the dataset. Fewer examples of severe and proliferative DR could have limited the models’ ability to learn representative features, which is a common challenge in medical image datasets [45]. Second, this study was conducted using a single dataset, which may restrict its generalizabilty across populations with different imaging conditions.

## 5. Conclusions

This study has presented a thorough comparative evaluation of two-state-of-the art CNN architectures, ResNeXt and RegNet, for the automated classification of diabetic retinopathy (DR) using the APTOS 2019 dataset. Our investigation encompassed both binary (DR vs non-DR) and multi-class (five-stage severity) classification tasks complemented by model interpretability through SHAP, thereby addressing both performance and transparency in clinical decision-making.

In binary classification, the ResNeXt101-64x4d model achieved the highest performance, with an AUC of 99.71% and an F1-score of 98%, underscoring its strong discriminatory ability and architectural robustness. The RegNetY32GT model demonstrated comparable predictive performance, achieving uniformly high scores across all metrics while maintaining lower architectural complexity and computational cost. This balance makes it a practical alternative for development in resource-constrained clinical settings.

For multi-class classification, ResNeXt101-32x8d and RegNetY16GT emerged as the top-performing models. Notably, RegNetY16GT demonstrated greater metric stability across intermediate and advanced DR stages, particularly in Class 2 (moderate) and Class 4 (proliferative), where diagnostic precision is clinically imperative. SHAP-based analyses indicate that both architectures focus on anatomically and pathologically meaningful retinal regions such as the macula, optic disc, and lesion clusters, providing model transparency that may support future clinical integration. Nonetheless, further validation by ophthalmologists is necessary to confirm the diagnostic relevance of these highlighted areas.

Overall, our findings suggest that ResNeXt and RegNet offer complementary strengths; ResNeXt models excel at capturing fine-grained features through deeper grouped convolutions, while RegNet variants achieve competitive performance with enhanced parameter efficiency and scalability. The integration of explainability tools enhances transparency and may support regulatory readiness and ethical deployment of AI-driven tools in ophthalmology; nonetheless, claims of clinical trustworthiness should be approached with caution until corroborated through expert validation and real-world clinical studies.

A noteworthy implication of this study is the demonstrated alignment between quantitative metrics and visual interpretability, which reinforces the validity of the model’s predictions in a medically meaningful context.

These findings highlight the solid performance of the evaluated architectures within the context of the APTOS 2019 dataset. However, it is important to emphasize that assessing the broader applicability and robustness of these models remains an open challenge. Future work will focus on evaluating model generalizability across independent datasets with varying imaging characteristics, acquisition protocols, and population distributions. This work is essential in order to verify whether the proposed models can maintain consistent performance beyond the APTOS 2019 dataset and adapt to real-world clinical variability. In addition, efforts will be directed towards addressing limitations such as class imbalance in severe DR stages by utilizing advanced data augmentation strategies (e.g., synthetic sample generation, reweighting schemes) and incorporating multimodal data sources to enrich model inputs, including patient demographics and optical coherence tomography (OCT) scans. Specifically, the pronounced drop in sensitivity and F1-score for Classes 3 and 4 highlights the need for targeted mitigation techniques. Future studies could explore loss function engineering and oversampling techniques in order to improve the representation of underrepresented classes during training. Evaluating the impact of these methods both independently and in combination will help to determine the optimal strategy for enhancing recognition of advanced DR stages without compromising performance in the more prevalent classes. Additionally, cross-dataset evaluation and prospective clinical validation are essential in order to assess generalizability and operational effectiveness in diverse real-world settings, including testing on datasets with varying DR prevalence rates and demographic profiles to verify the robustness and fairness of model predictions across patient subgroups.

## Figures and Tables

**Table 1:** Overview of recent DL-based approaches for DR classification.

Author	CNN Architectures	Dataset	Best Model and Performance
Mostafa et al. [20]	DenseNet201, ResNet50, VGG19 MobileNetV2, GCN (ensemble)	APTOS 2019	MobileNetV2+GCN (82.5% accuracy, AUC = 0.88)
Herrero et al. [21]	DenseNet, InceptionV3, MobileNetV3 ResNet50 (modified), VGG, Xception	APTOS 2019, EyePACS, DDR, IDRiD, SUSTech-SYSU	Modified ResNet50 (94.64% accuracy, AUC = 0.98, QWK = 0.94)
Kumar and Ravi [27]	Inception-ResNet-v2 + Custom CNN	APTOS 2019, Messidor-1	Hybrid Inception-ResNet-v2 (82.18% accuracy, on APTOS 2019)
Ai et al. [29]	InceptionV3, InceptionResNetV2, Xception, ResNeXt101, NASNetLarge (ensemble)	Kaggle Diabetic Retinopathy	DR-IIXRN (ensemble model) (accuracy: 0.79, no-DR: F1-score = 0.89, PDR: F1-score = 0.6)
Ramchandre et al. [30]	SEResNeXt32x4d, EfficientNetB3	APTOS 2019	EfficientNetB3 (91.44% accuracy)
Youldash et al. [31]	EfficientNetB3, EfficientNetV2B1, RegNetX008, RegNetX080, RegNetY006, RegNetY008	APTOS 2019, AI-Saif Medical Center	RegNetX080 (binary: 98.6% accuracy), EfficientNetB3 (multiclass: 85.1% accuracy)

**Table 2:** Class distribution of DR grades across training, validation, and test sets (multi-class classification).

	Classes				
Subset	0	1	2	3	4
Train	1264	260	700	136	207
Validation	362	76	201	39	60
Test	182	38	101	21	31

**Table 3:** Distribution of samples for binary DR classification across training, validation, and test subsets.

	Classes	
Subset	0	1
Train	1264	1301
Validation	361	373
Test	183	186

**Table 4:** Preprocessing transformations applied to APTOS dataset.

Name	Description	Values
Input	Image size	(224, 224)
ColorJitter	Random adjustment of brightness, contrast, saturation, and hue of the image	Brightness: 0.05 Contrast: 0.05 Saturation: 0.05 Hue: 0.05
RandomHorizontalFlip	Randomly flips the image horizontally	-
RandomVerticalFlip	Randomly flips the image vertically	-
RandomRotation	Randomly rotates the image within a specified range	Degrees: 20
Normalize	Normalizes the image with specific means and standard deviations for the RGB color channels	Mean: [0.485, 0.456, 0.406] Std: [0.229, 0.224, 0.225]

**Table 5:** Summary of the computational characteristics of the utilized CNN architectures.

Architecture	Total Parameters	GFLOPs	Inference Time (ms)	GPU Memory (MB)
ResNeXt50-32x4d	25,028,904	4.29	7.96	214.68
ResNeXt101-32x8d	88,791,336	16.54	14.23	689.14
ResNeXt101-64x4d	83,455,272	15.58	13.86	1007.86
RegNetX32GF	107,811,560	31.88	13.32	1475.36
RegNetY16GF	83,590,140	16.01	15.48	1708.09
RegNetY32GF	145,046,770	32.41	15.77	2363.20

**Table 6:** Training configuration and hyperparameter settings used for model development.

Configuration	Details
Data splitting	Training (70%), Validation (20%), Testing (10%)
Optimizer	Adam optimizer with an initial learning rate of 0.001
Loss function	Cross-Entropy Loss
Batch size	32
Epochs	30

**Table 7:** Performance metrics of ResNeXt and RegNet architectures for binary DR classification.

Model	Class	AUC (%)	Precision (%)	Sensitivity (%)	Specificity (%)	F1-Score (%)
ResNeXt50-32x4d	0	99.34	95	97	94.59	96
	1	99.34	97	95	97.25	96
ResNeXt101-32x8d	0	99.37	94	97	94.05	95
	1	99.37	97	94	94.67	95
ResNeXt101-64x4d	0	99.71	96	99	95.68	98
	1	99.71	99	96	99.45	98
RegNetX32GT	0	99.49	97	96	99.70	96
	1	99.49	96	97	96.22	96
RegNetY16GT	0	99.48	96	96	95.60	96
	1	99.48	96	96	95.68	96
RegNetY32GT	0	99.31	97	97	97.25	97
	1	99.31	97	97	96.76	97

**Table 8:** Multi-class classification performance metrics for ResNeXt and RegNet models.

Model	Metric	Class 0	Class 1	Class 2	Class 3	Class 4
ResNeXt50-32x4d	AUC	99.77	91.18	92.62	92.07	92.50
	Precision	95	70	67	57	73
	Sensitivity	99	38	92	20	37
	Specificity	94.65	98.19	83.21	99.14	98.82
	F1-score	97	49	78	30	49
ResNeXt101-32x8d	AUC	99.84	91.46	92.56	92.35	88.86
	Precision	96	58	70	54	60
	Sensitivity	98	38	88	35	40
	Specificity	95.72	96.98	86.19	98.28	97.63
	F1-score	97	46	78	42	48
ResNeXt101-64x4d	AUC	99.23	90.32	93.22	92.69	87.86
	Precision	93	80	69	35	64
	Sensitivity	99	32	86	35	30
	Specificity	92.51	99.09	85.45	96.26	98.52
	F1-score	96	46	76	35	41
RegNetX32GT	AUC	99.76	91.71	91.13	91.88	91.95
	Precision	95	53	64	67	52
	Sensitivity	98	24	81	30	47
	Specificity	94.65	97.58	82.84	99.14	96.15
	F1-score	96	33	71	41	49
RegNetY16GT	AUC	99.59	90.84	93.25	89.24	92.96
	Precision	98	72	73	35	57
	Sensitivity	98	35	89	30	57
	Specificity	97.86	98.49	87.69	96.84	96.15
	F1-score	98	47	80	32	57
RegNetY32GT	AUC	99.58	92.34	91.88	91.55	92.88
	Precision	95	48	72	100	55
	Sensitivity	98	59	76	5	53
	Specificity	95.19	95.78	88.81	100	96.15
	F1-score	96	53	74	10	54

**Table 9:** Best-performing models from the ResNeXt and RegNet families for binary and multi-class DR classification.

Task	Model	Class	AUC (%)	Precision (%)	Sensitivity (%)	Specificity (%)	F1-Score (%)
Binary	ResNeXt101-64x4d	0	99.71	96	99	95.68	98
		1	99.71	99	96	99.45	98
	RegNetY32GT	0	99.31	97	97	97.25	97
		1	99.31	97	97	96.76	97
Multi-class	ResNeXt101-32x8d	0	99.84	96	98	95.72	97
		1	91.46	58	38	96.98	46
		2	92.56	70	88	86.19	78
		3	92.35	54	35	98.28	42
		4	88.86	60	40	97.63	48
	RegNetY16GT	0	99.59	98	98	97.86	98
		1	90.84	72	35	98.49	47
		2	93.25	73	89	87.69	80
		3	89.24	35	30	96.84	32
		4	92.96	57	57	96.15	57

**Table 10:** Class-wise performance comparison.

Model	DR Grade	Precision (%)	Recall (%)	F1-Score (%)
ResNeXt101-32x8d (Proposed)	0	96	98	97
	1	58	38	46
	2	70	88	78
	3	54	35	42
	4	60	40	48
DL model (Ayala et al. [42])	0	96	99	97
	1	73	49	59
	2	70	84	77
	3	40	26	31
	4	46	36	40

## Data Availability

The data presented in this study are available in Kaggle at https://www.kaggle.com/c/aptos2019-blindness-detection/overview, (accessed on 1 July 2024).
